# Treatment-associated imaging changes in newly diagnosed *MGMT* promoter-methylated glioblastoma undergoing chemoradiation with or without cilengitide

**DOI:** 10.1093/neuonc/noad247

**Published:** 2024-01-14

**Authors:** Christina Maria Flies, Michel Friedrich, Philipp Lohmann, Karin Alida van Garderen, Marion Smits, Joerg-Christian Tonn, Michael Weller, Norbert Galldiks, Tom Jan Snijders

**Affiliations:** Department of Neurology & Neurosurgery, UMC Utrecht Brain Center, University Medical Center Utrecht, Utrecht, The Netherlands; Institute of Neuroscience and Medicine (INM-3, INM-4), Research Center Juelich, Juelich, Germany; Institute of Neuroscience and Medicine (INM-3, INM-4), Research Center Juelich, Juelich, Germany; Department of Radiology & Nuclear Medicine, Erasmus MC, University Medical Centre Rotterdam, Rotterdam, The Netherlands; Brain Tumour Centre, Erasmus MC Cancer Centre, Rotterdam, The Netherlands; Medical Delta, Delft, The Netherlands; Department of Radiology & Nuclear Medicine, Erasmus MC, University Medical Centre Rotterdam, Rotterdam, The Netherlands; Brain Tumour Centre, Erasmus MC Cancer Centre, Rotterdam, The Netherlands; Medical Delta, Delft, The Netherlands; Department of Neurosurgery, University Hospital Munich LMU, Munich, Germany; Department of Neurology, Clinical Neuroscience Center, University Hospital and University of Zurich, Zurich, Switzerland; Institute of Neuroscience and Medicine (INM-3, INM-4), Research Center Juelich, Juelich, Germany; Department of Neurology, Faculty of Medicine and University Hospital Cologne, University of Cologne, Cologne, Germany; Center for Integrated Oncology (CIO), Universities of Aachen, Bonn, Cologne, and Duesseldorf, Cologne, Germany; Department of Neurology & Neurosurgery, UMC Utrecht Brain Center, University Medical Center Utrecht, Utrecht, The Netherlands

**Keywords:** glioma, modified RANO criteria, pseudoprogression, temozolomide

## Abstract

**Background:**

Radiological progression may originate from progressive disease (PD) or pseudoprogression/treatment-associated changes. We assessed radiological progression in O^6^-methylguanine-DNA methyltransferase (*MGMT*) promoter-methylated glioblastoma treated with standard-of-care chemoradiotherapy with or without the integrin inhibitor cilengitide according to the modified response assessment in neuro-oncology (RANO) criteria of 2017.

**Methods:**

Patients with ≥ 3 follow-up MRIs were included. Preliminary PD was defined as a ≥ 25% increase of the sum of products of perpendicular diameters (SPD) of a new or increasing lesion compared to baseline. PD required a second ≥25% increase of the SPD. Treatment-associated changes require stable or regressing disease after preliminary PD.

**Results:**

Of the 424 evaluable patients, 221 patients (52%) were randomized into the cilengitide and 203 patients (48%) into the control arm. After chemoradiation with or without cilengitide, preliminary PD occurred in 274 patients (65%) during available follow-up, and 88 of these patients (32%) had treatment-associated changes, whereas 67 patients (25%) had PD. The remaining 119 patients (43%) had no further follow-up after preliminary PD. Treatment-associated changes were more common in the cilengitide arm than in the standard-of-care arm (24% vs. 17%; relative risk, 1.3; 95% CI, 1.004–1.795; *P* = .047). Treatment-associated changes occurred mainly during the first 6 months after RT (54% after 3 months vs. 13% after 6 months).

**Conclusions:**

With the modified RANO criteria, the rate of treatment-associated changes was low compared to previous studies in *MGMT* promoter-methylated glioblastoma. This rate was higher after cilengitide compared to standard-of-care treatment. Confirmatory scans, as recommended in the modified RANO criteria, were not always available reflecting current clinical practice.

**Key Points**

- Using the 2017 RANO criteria, 32% had confirmed treatment-associated changes.
- Cilengitide significantly increased the rate of treatment-associated changes.

**Importance of the Study**

Response evaluation in irradiated gliomas remains difficult due to treatment-associated changes. A modification of the 2010 response assessment in neuro-oncology (RANO) criteria was proposed in 2017 to improve radiological response assessment. We reviewed a large, multicenter cohort of patients with O^6^-methylguanine-DNA methyltransferase (*MGMT*) promoter-methylated glioblastoma treated with standard chemoradiation with or without cilengitide. With modified RANO, we found a low rate of treatment-associated changes (21%) compared to previous studies, which in part may be related to the stringent criteria. Eighty-eight of 274 patients with progression (32%) had treatment-associated changes. The interpretation was difficult in 119 patients (43%) because no confirmatory scan was made after preliminary PD.

The rate of treatment-associated changes was highest during the first 3 months after RT and higher after cilengitide than after standard-of-care treatment.

Our findings may facilitate further research to better understand the pathophysiology of treatment-associated changes. This study may offer practical insights for the design of future clinical trials.

Diffuse gliomas are the most common adult-onset malignant primary brain tumors.^1,2^ Despite extensive treatment, median survival for aggressive CNS WHO grade 4 glioblastomas is only 12–18 months.^1^ The standard-of-care treatment includes maximum safe resection and radiotherapy (RT) with concomitant and maintenance chemotherapy with temozolomide.^3^ Patients whose glioblastoma harbors methylation of the O^6^-methylguanine-DNA methyltransferase (*MGMT*) promoter show higher response rates to temozolomide than those with an unmethylated *MGMT* promoter.^4^

Cilengitide is an integrin inhibitor with antiangiogenic activity and was used in clinical trials combined with standard treatment, both for *MGMT* promoter-methylated and unmethylated glioblastomas.^5,6^ No survival benefit was found with the addition of cilengitide in either group.

Radiological and/or clinical worsening may be seen at any time posttreatment and may be the result of actual progressive disease (PD) or an effect of treatment-associated changes. Early, self-limiting pseudoprogression and late, more progressive radiation necrosis are the most prominent subtypes within the spectrum of such treatment-associated changes.^7–11^

Angiogenesis inhibitors, such as cilengitide or bevacizumab, are supposed to block the formation of new vessels. Bevacizumab restores the permeability of leaky vessels through blocking effects on vascular endothelial growth factor (VEGF) signaling. In the large prospective AVAglio trial comparing standard treatment with bevacizumab to standard treatment with placebo in glioblastomas, considerably fewer patients in the intervention arm developed pseudoprogression compared to the control arm (2.2% vs. 9.3%).^12^ The effect of cilengitide on the rate of treatment-associated changes has not yet been investigated.

The response assessment in neuro-oncology (RANO) criteria of 2010 provided radiological recommendations to assess treatment response on anatomical MRI. In these criteria, the most common method to diagnose PD required an increase of ≥25% in the sum of products of the perpendicular diameters (SPD) of enhancing lesions.^13^ Because of the probability of early pseudoprogression, PD cannot be diagnosed in the first 3 months postradiation.

The proposal for modified RANO criteria for radiographic response assessment in glioblastoma of 2017 added an interval of 4 weeks or more between the first increase for preliminary PD and a second increase of ≥25% for confirmed PD.^14^ In the case of no further increase, but rather stable or regressing disease, confirmed pseudoprogression is diagnosed. This proposed change from the 2010 criteria reflects the insight that treatment-associated imaging changes can occur beyond three months postradiation. Another suggestion was to use the first postradiation MRI and not the early postoperative MRI as the baseline MRI, because of possible postsurgical artifacts and timing, as well as protocol variations between centers of the postsurgery MRI as patients are not yet included in clinical trials.

The main objective of this study was to subdivide new or increasing contrast-enhancing (CE) lesions into PD versus treatment-associated changes using the modified RANO criteria.^14^ Specifically, we aimed to determine the incidence of treatment-associated imaging changes as a function of: (a) timing after treatment and (b) the addition of cilengitide.

## Methods

The EORTC-CENTRIC study (NCT00689221) was a multicenter, open-label, phase-3 randomized clinical trial to investigate the effect of cilengitide on overall survival (OS) in patients with *MGMT* promoter-methylated glioblastoma; in this trial, the addition of cilengitide to standard-of-care chemoradiation did not result in an OS benefit. Details on the trial design and its main results have been published.^5^ Informed consent was available from all participants for the initial study. As stated in the original paper, the CENTRIC study was approved by the institutional review boards or independent ethics committees of the participating institutions and competent authorities according to country-specific regulations.^5^ The current study is a secondary analysis of the imaging data and the original consent also covers additional studies of the imaging data.

For this retrospective study of a prospectively collected cohort, 2 clinical researchers (CMF and MF) reviewed 60% and 40%, respectively, of the MRIs of the CENTRIC database to determine the rates of PD and treatment-associated changes according to the modified RANO criteria.^14^ The first 10 patients were assessed by both readers and then discussed in the group (CMF, MF, TJS, NG, PL) to test the agreement between the 2 raters and verify the radiological criteria. All cases wherein the reader was uncertain about the correct evaluation were discussed in this same group.

### Inclusion Criteria

All patients with the availability of: (a) 3 or more follow-up MRIs in total and at least 2 after the end of RT, and (b) all relevant clinical information, were included. Relevant clinical data consisted of data on treatment allocation, RT, progression-free survival (PFS), and second-line therapy.

We hypothesized that the excluded patients with missing data showed early progression and examined if their time from diagnosis to the PFS date was less than three months. Furthermore, we noted their treatment allocation group.

### Definition of Baseline, Response, and Outcome

As proposed in the modified RANO criteria, the baseline MRI was defined as the first MRI after the end of RT.^14^ In case of a (partial or complete) response during follow-up, the best response MRI before preliminary PD was defined as the new nadir and the new baseline MRI.

To detect early pseudoprogression, we also compared the first MRI after RT (baseline MRI as defined before) to the pre-RT MRI. In the case of preliminary PD on the first MRI after RT compared to the pre-RT MRI and no postsurgical artifacts that hindered the evaluation, the pre-RT MRI was defined as the baseline MRI.

**Figure 1.**
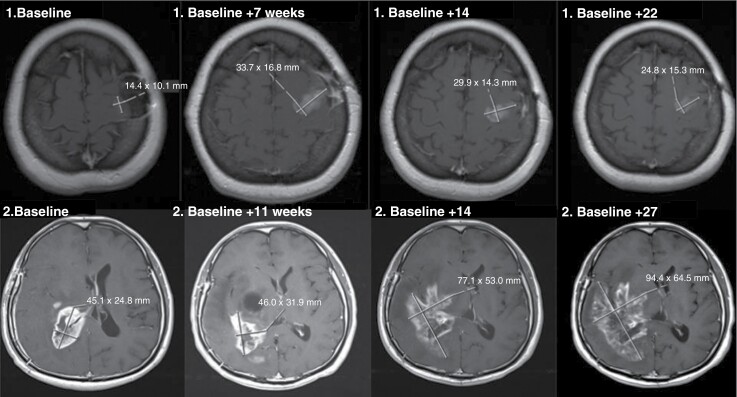
Illustration of treatment-associated changes and progressive disease. Top row 1: Patient example for treatment-associated changes. On the first MRI (1. baseline), the baseline scan was performed 3 weeks after the end of radiotherapy. The first follow-up MRI 7 weeks later (1. baseline + 7 weeks) shows preliminary progressive disease. The 2 follow-up MRIs show a decrease after 14 weeks (1. baseline + 14) compared to “1. Baseline+7 weeks” and a stable lesion after 22 weeks (1. baseline + 22). This patient was treated with cilengitide in addition to standard therapy. Bottom row 2: Patient example for progressive disease. The baseline MRI (2. baseline) was performed 2 weeks before the start of radiotherapy. This patient developed preliminary progressive disease on the first MRI after radiotherapy 11 weeks later (2. baseline + 11 weeks). The 2 follow-up MRIs show further progression of the lesion at weeks 14 and 27 (2. baseline + 14 and 2. baseline + 27). This patient was part of the control group without cilengitide.

Preliminary PD was defined as a first ≥25% increase of the SPD of the contrast-enhancing lesion compared to baseline on the axial T1-weighted MRI after gadolinium-based contrast agent administration. The outcome PD was defined as a second ≥25% increase of the SPD on at least 1 follow-up MRI four weeks later. A new measurable lesion outside of the radiation field was immediately considered to be PD according to the 2010 RANO criteria.^13^ A new measurable lesion within the radiation field was added to the SPD until a maximum of five lesions. The outcome of “treatment-associated changes” was defined as either stable disease (SD, <25% increase or <50% decrease on at least 2 follow-up MRIs, each 1 at least 4 weeks after the previous); or partial or complete response (>50% decrease on at least 1 follow-up MRI 4 weeks later) after preliminary PD. Figure 1 depicts an example of PD and treatment-associated changes.

The response included partial or complete response (PR/CR), preliminary partial or complete response (pPR, pCR) or pseudoresponse (PsR) as defined by the modified RANO criteria.^14^ We classified the outcomes of patients with no RANO progression within the available follow-up time as (preliminary) partial response (pPR, PR), (preliminary) complete response (pCR, CR), stable disease (SD), or pseudoresponse (PsR). The best response of patients with no measurable disease at baseline was SD. For patients who did not have further follow-up MRIs after preliminary PD, we classified the outcome as unconfirmed PD.

Certain cases could not be classified according to the above-mentioned criteria since there was only 1 follow-up scan after preliminary PD without a clear increase or decrease. To provide a meaningful classification for these patients and to prevent the formation of a biased set of “unclassifiable” cases, we handled these cases as follows: in the case of only 1 follow-up scan at least eight weeks after the preliminary PD scan showing SD (<15% increase) and the date of PFS was not the date of the preliminary PD scan, then we classified the course as treatment-associated changes. In the case of only 1 follow-up scan fewer than 8 weeks later and/or the lesion increased >15% and/or the PFS date corresponded to the preliminary PD date, then the outcome was defined as unconfirmed PD. In these cases, true progression was suspected but the modified RANO criteria for PD were not met.

A new treatment introduced before the end of available follow-up could influence the outcome. Therefore, we excluded patients with an outcome defined as SD or treatment-associated changes who started a second treatment before the last available scan (*n* = 6). Salvage surgery on a lesion that developed on the PFS date was regarded as a second treatment. Otherwise, a second surgery was ignored. All lesions that were labeled as “(probably) outside the radiation field” were reviewed by an experienced neuro-oncologist (TJS). Of note, planning information on radiation fields was not available for comparison; we estimated the extent of the radiation field from the T2-weighted FLAIR hyperintense and contrast-enhancing regions on postoperative MRI. Difficult cases, and problems with the use of the response criteria, were discussed and solved in consensus meetings between the first 2 authors (MF, CMF) and the 2 senior authors (NG, TJS).

We evaluated the MRIs according to the modified RANO criteria. The most used criteria in previous clinical trials are however the RANO criteria of 2010, which served also in the CENTRIC study.^13^ To compare the results based on the modified RANO criteria of 2017 with the RANO criteria of 2010, we also give an overview of the results according to the 2010 RANO criteria. Table 1 summarizes the main differences between the 2010 and 2017 RANO criteria.

**Table 1.** Summary of the 2010 RANO and 2017 Modified RANO Criteria

Criterion	2010 RANO^13^	2017 Modified RANO^14^
Baseline	Postoperative scan	Postradiation scan
Progressive disease	≥25% increase of the SPD	2× ≥ 25% increase of the SPD
Treatment-associated changes	Not defined	≥25% increase of the SPD followed by SD, PR or CR
Response	>50% decrease in SPD (PR) Complete disappearance of all enhancing lesions (CR)	>50% decrease in SPD followed by SD or further decrease (PR) Sustained complete disappearance of all enhancing lesions (CR)
Confirmatory scan required	No	Yes

SPD =  sum of products of perpendicular diameters; SD = stable disease, PR = partial response, CR = complete response.

### Analysis

Time-to-progression (TTP) was defined as the time from the end of RT to preliminary PD and categorized into 3 subgroups: before 3, from 3 to 6, and after 6 months after RT. We calculated a relative risk with a 95% CI and *P*-value for the development of treatment-associated changes in the cilengitide group compared to the control group. We used a chi-square test with 95% CI and *P*-value to compare the patients with missing or no missing values between the allocation groups. To compare the survival curves of patients with PD and treatment-associated changes, the log-rank test was used.

Continuous variables were presented as median or mean with an interquartile range (IQR) or SD. SPSS version 26.0.0.1 (2019, IBM SPSS Statistics, Armonk, USA) and MedCalc version 20.019 (2021, MedCalc Software Ltd, Ostend, Belgium) were used for the calculations. Two-tailed *P*-values < .05 were considered statistically significant.

## Results

In total, we reviewed 4010 MRIs from 545 patients randomized into the CENTRIC study. The patient characteristics are given in the original article.^5^ In brief, men were the predominant group (53%), the median age was 58 years, and almost half had undergone a gross total resection (49%). Of all patients, 49.9% were randomized into the cilengitide arm, and 50.1% in the control arm. Due to missing MRIs or clinical data, 121 patients were excluded from the analysis. Of the remaining 424 patients, 221 patients (52%) had been randomized into the cilengitide arm and 203 patients (48%) into the control arm. The median SPD at baseline or nadir was 0 mm^2^; interquartile range (IQR), 565.3 (0–565.3). RANO measurable disease at baseline or nadir was present in 283 patients (67%). RANO response led to a new baseline MRI in 102 patients (24%). In total, 141 patients (33%) had no measurable disease at baseline.

After chemoradiation with or without cilengitide, preliminary PD occurred in 274 patients (65%) after a median TTP of 5.2 months (IQR 11.9 (2.3–14.2)), and 3.6 months after baseline or nadir (IQR 6.5 (2.2–8.7)) with a median SPD of 674.9 mm^2^ (IQR 1012.5 (351.2-1363.7)). After a follow-up of these 274 patients, treatment-associated changes were diagnosed in 88 patients (32%) and PD was diagnosed in 67 patients (25%). The remaining 119 patients (43%) had no further follow-up MRI after preliminary PD and were defined as unconfirmed PD, mostly because a clinical decision to diagnose PD was made. The overall rate of treatment-associated changes was 21% (88 of 424 patients).

Seven of the 15 patients with only 1 follow-up MRI after preliminary PD were classified as treatment-associated changes and 8 as unconfirmed PD.

**Figure 2.**
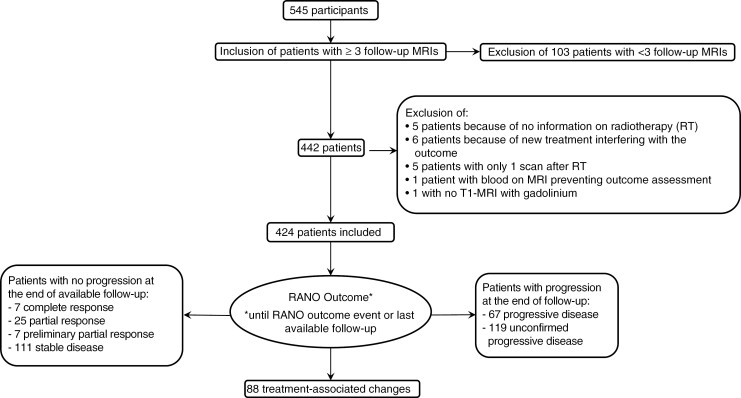
Flowchart of the inclusion process and outcome categories.

We observed a response to treatment in 101 patients, mostly partial response (*n* = 61). The outcome analysis showed no progression within the available follow-up time in 150 patients (35% of 424 patients). Figure 2 depicts the patient selection process and the detailed outcome groups.

**Figure 3.**
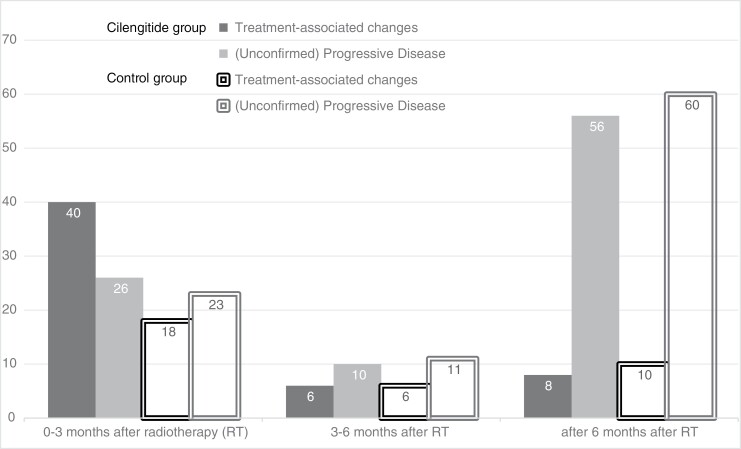
Outcome after preliminary progressive disease, depicted per time period and per treatment group (cilengitide or control arm). Time was measured from the end of radiotherapy (RT) to preliminary progressive disease. The group “(Unconfirmed) Progressive Disease” includes the outcome groups with unconfirmed progressive disease (who were mostly considered clinically to have progressive disease), and those with progressive disease according to modified RANO.

Figure 3 shows the outcome after preliminary PD for different time periods. Of all patients who developed preliminary PD 3 months after RT, 54% were diagnosed with treatment-associated changes and 46% with PD or unconfirmed PD. However, of all the patients with preliminary PD after 6 months after RT, 13% had treatment-associated changes, and 87% had PD or unconfirmed PD.

In the cilengitide group of 221 patients, 54 patients (24%) developed treatment-associated changes, and 30 patients (14%) developed PD, whereas in the control group of 203 patients, 34 patients (17%) developed treatment-associated changes, and 37 patients (18%) PD. The relative risk for the development of treatment-associated changes in the cilengitide group compared to the control group was 1.3 (95% CI 1.004–1.795; *P* = .047).

Five patients were diagnosed with PD based on new measurable lesions outside of the radiation field.

### Patients with Missing Data

The progression date (PFS) of 100 of the 121 excluded patients occurred within 3 months after diagnosis and after 3 months in another 21 patients. Numerically, more excluded patients had been randomized into the control group than into the intervention group (70 vs. 51 patients, chi-square test 3.8, *P* = .05).

### Radiological Criteria

Here we give an overview of the results according to the 2010 RANO criteria: Progressive disease within the first 12 weeks following the end of RT can only be diagnosed in the case of a new lesion outside of the radiation field or by neuropathological confirmation. Therefore, we would have had to wait for further follow-up in 106 of 107 patients who progressed in our study within the first three months after RT before diagnosing progression or treatment-associated changes. In the last patient, a new lesion outside of the radiation field was found and progression could have been called. All 167 patients with preliminary PD after 12 weeks after RT (those with treatment-associated changes, PD and unconfirmed PD) would have been diagnosed with PD, without the need for a confirmatory scan. This difference in response assessment may also explain why we found no progression in 35% of patients in our study, although a PFS date was noted for these patients in the original CENTRIC data.

### Survival

**Figure 4.**
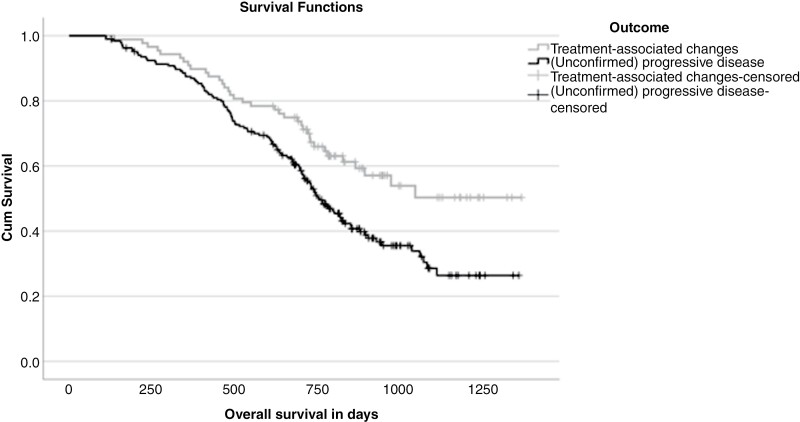
Kaplan–Meier curve to compare the survival of patients with treatment-associated changes and those with (unconfirmed) progressive disease. The *P*-value of the log-rank test was .003.

The mean overall survival (OS) of the patients according to modified RANO with treatment-associated changes was 997.1 days/32.8 months (95% CI 905.0–1089.2) and for those with (unconfirmed) progressive disease 822.2 days/27.0 months (95% CI 760.6–883.7). The *P*-value of the log-rank test was 0.003. The Kaplan–Meier curve is included in Figure 4. The mean OS of patients with progressive disease (with a confirmatory scan) was 877.8 days/28.8 months (95% CI 775.2–980.4). The comparison with those with treatment-associated changes produced a *P*-value of .115 (log-rank test). We supposed that the patients with unconfirmed PD showed clear clinical progression and therefore did not undergo a confirmatory scan. We compared the OS of the patients with unconfirmed and confirmed PD, which showed a numerical lower OS for the patients with unconfirmed PD (mean OS 766.8 days/25.2 months) (95% CI 698.8–834.7), *P*-value of log-rank test .151).

## Discussion

In this retrospective analysis of a multicenter, open-label, phase-3 randomized clinical trial in patients with *MGMT* promoter-methylated glioblastoma treated with cilengitide, we reviewed MRI data from 545 patients according to the modified RANO criteria. After the exclusion of patients with missing data, we found an increased risk for treatment-associated changes in the cilengitide group (24% of 221 patients) compared to the control group (17% of 203 patients). These numbers produced a relative risk of 1.3 (95% CI = 1.004–1.795).

Our rate of treatment-associated changes (21%) is comparable to previous literature in which incidences in patients with glioblastoma treated with temozolomide-based chemoradiation between 3% and 31% were described.^15–21^ However, the rate in patients with an *MGMT* promoter-methylated glioblastoma was generally higher in earlier studies with percentages reaching 31–58%.^15–18^ This discrepancy between our rate and previous rates could be the consequence of the use of the more stringent, modified RANO criteria in our study with the necessity to await a confirmatory scan before diagnosing treatment-associated changes. The previously mentioned author groups assessed tumor progression according to the Macdonald’s, the 2010 RANO, or their own criteria.^13,22^

Furthermore, the incidence of treatment-associated changes may be lower in the treatment arm due to a similar antiangiogenic effect of cilengitide to that of bevacizumab in previous studies.^12^ We found the contrary: a higher incidence in the treatment arm. Of note, cilengitide has a different mechanism of action compared to bevacizumab with a less prominent, if any, effect on the blood–brain barrier.^6,23^ Local hypoxia in tumors leads to the binding of hypoxia-inducible factor to the vascular endothelial growth factor (VEGF) gene and the release of circulating VEGF. VEGFs bind to VEGF-receptors (VEGFR) on endothelial cells, which stimulates their aggregation and proliferation, and leads to the expression of integrins. Integrins expressed in various cell types enable the cells to link to extracellular matrix proteins and to migrate and form for example new vessels. Bevacizumab binds to VEGF and hinders the linkage to the VEGFR-1 and 2, whereas the integrin inhibitor cilengitide blocks the linkage of the integrins αvβ3 and αvβ5 to ligands or matrix proteins.^6,23,24^ It has commonly been assumed that bevacizumab restores the permeability of the blood–brain barrier leading to a reduction of T1-contrast enhancement and T2-vasogenic edema seen in treatment-associated changes, which both at least partly depend on VEGF.^25–30^ Integrins αvβ3 and αvβ5 interact with VEGFR-2 on endothelial (tumor) cells,^31–34^ whereas VEGFR-1 interacts with β1 integrins.^35^ A higher incidence of treatment-associated changes after cilengitide compared to standard treatment may be explained by a transient normalization of the blood–brain barrier by cilengitide improving perfusion and an in vivo observed increase in VEGFR-2-dependent angiogenesis.^5,30,34,36^ Considering, therefore, the VEGF-pathway, αvβ3 and αvβ5 blockage could lead to more imaging changes related to treatment-associated changes compared to a blockage of VEGFR-1 and 2, because the development of treatment-associated changes may rely more on VEGFR-1 and the development of pseudoresponse may depend on VEGFR-1 blocking mechanisms.

Treatment-associated changes developed mostly within three months after RT (66% of all treatment-associated changes; 54% of all patients). In earlier studies, 12–64% of all patients with treatment-associated changes had these changes within 3 months after RT.^15,16,18–21^

The limitations of our study are the retrospective nature, the single assessment of a part of the database by 2 nonradiologists and no information on the radiation fields. However, the exact radiation fields were only necessary in the small number of patients (*n* = 5) with a probable out-of-field lesion. This limitation is therefore considered negligible. To ensure the quality of the assessment, the researchers were trained by 2 experienced neuro-oncologists. In addition, repeated consensus meetings for the review of cases with these researchers and neuro-oncologists were held. Finally, additional discussions with a European expert panel on oncological neuroradiology, the EORTC Brain tumor imaging committee, took place. The retrospective design led to 43% unconfirmed PD outcomes with no further follow-up, because the original study used the 2010 RANO criteria for outcome evaluation and, consequently, did not require a confirmatory scan for increasing MRI lesions.^5^ However, in the most recent RANO recommendations, a confirmatory scan is advised in the first 3 months after radiotherapy, but not mandatory; at later time points, it is not advised.^37^ In our data, we saw that the patients with “unconfirmed PD” (i.e. without a confirmatory scan) had survival that was numerically worse than for “confirmed PD,” supporting the notion that most cases of “unconfirmed PD”-cases were true PD.

We did not add volumetric data as the dataset was too heterogeneous—and thus insufficient—for efficient and robust methods of automated volume segmentation. Adding volumetric data in future studies could extend and expand our findings.

The prespecified assessment criteria and the large, multicenter, multinational sample constitute the strengths of this study. The consensus meetings and the close supervision by 2 experienced neuro-oncologists (TJS, NG) increased the interrater reliability of the rating.

This study illustrates the constraints of the 2010 RANO criteria.^13^ The lack of a clear definition of treatment-associated changes forces clinicians to rely on clinical status and experience to make a diagnosis, and therefore many different definitions of treatment-associated changes are described in the literature. In this study, 119 patients were diagnosed with unconfirmed PD in the absence of a confirmatory scan. If different criteria were used, such as the 2010 RANO criteria, they might have been diagnosed as progressive. Some of these 119 may have had treatment-associated changes, as we have seen that 88 patients in this study were diagnosed as such after initial preliminary PD.

In conclusion, the rate of treatment-associated changes in the CENTRIC database was low compared to previous studies in newly diagnosed *MGMT* promoter-methylated glioblastoma. This could be the consequence of the stringent diagnostic criteria used. We observed more treatment-associated changes after cilengitide compared to standard treatment alone. The added value of new criteria sets such as the proposed modified RANO criteria of 2017 needs to be validated, ideally in a prospective setting.

## Data Availability

No new data were generated or analyzed in support of this research.

## References

[1] Louis DN , Perry A, Reifenberger G, et al. The 2016 World Health Organization classification of tumors of the central nervous system: a summary. Acta Neuropathol. 2016;131(6):803–820. 27157931 10.1007/s00401-016-1545-1

[2] Ostrom QT , Cioffi G, Gittleman H, et al. CBTRUS statistical report: primary brain and other central nervous system tumors diagnosed in the United States in 2012–2016. Neuro Oncol. 2019;21(Suppl 5):v1–v100. 10.1093/neuonc/noz150 PMC6823730 31675094

[3] Stupp R , van den Bent MJ, Weller MF, et al. Radiotherapy plus concomitant and adjuvant temozolomide for glioblastoma. N Engl J Med. 2005;352(10):987–996. 15758009 10.1056/NEJMoa043330

[4] Hegi ME , Diserens AC, Gorlia T, et al. MGMT gene silencing and benefit from temozolomide in glioblastoma. N Engl J Med. 2005;352(10):997–1003. 15758010 10.1056/NEJMoa043331

[5] Stupp R , Hegi ME, Gorlia T, et al.; European Organisation for Research and Treatment of Cancer (EORTC). Cilengitide combined with standard treatment for patients with newly diagnosed glioblastoma with methylated MGMT promoter (CENTRIC EORTC 26071-22072 study): a multicentre, randomised, open-label, phase 3 trial. Lancet Oncol. 2014;15(10):1100–1108. 25163906 10.1016/S1470-2045(14)70379-1

[6] Reardon DA , Cheresh DC. Cilengitide: a prototypic integrin inhibitor for the treatment of glioblastoma and other malignancies. Genes Cancer. 2011;2(12):1159–1165. 22866207 10.1177/1947601912450586 PMC3411133

[7] Brandsma D , Stalpers L, Taal W, Sminia P, van den Bent MJ. Clinical features, mechanisms, and management of pseudoprogression in malignant gliomas. Lancet Oncol. 2008;9(5):453–461. 18452856 10.1016/S1470-2045(08)70125-6

[8] Verma N , Cowperthwaite MC, Burnett MG, Markey MK. Differentiating tumor recurrence from treatment necrosis: a review of neuro-oncologic imaging strategies. Neuro Oncol. 2013;15(5):515–534. 23325863 10.1093/neuonc/nos307 PMC3635510

[9] Ellingson BM , Chung C, Pope WB, Boxerman JL, Kaufmann TJ. Pseudoprogression, radionecrosis, inflammation or true tumor progression? Challenges associated with glioblastoma response assessment in an evolving therapeutic landscape. J Neurooncol. 2017;134(3):495–504. 28382534 10.1007/s11060-017-2375-2 PMC7893814

[10] Walker AJ , Ruzevick J, Malayeri AA, et al. Postradiation imaging changes in the CNS: how can we differentiate between treatment effect and disease progression? Future Oncol. 2014;10(7):1277–1297. 24947265 10.2217/fon.13.271 PMC4325371

[11] Winter SF , Loebel F, Loeffler J, et al. Treatment-induced brain tissue necrosis: a clinical challenge in neuro-oncology. Neuro Oncol. 2019;21(9):1118–1130. 30828724 10.1093/neuonc/noz048 PMC7594558

[12] Wick W , Chinot OL, Bendszus M, et al. Evaluation of pseudoprogression rates and tumor progression patterns in a phase III trial of bevacizumab plus radiotherapy/temozolomide for newly diagnosed glioblastoma. Neuro Oncol. 2016;18(10):1434–1441. 27515827 10.1093/neuonc/now091 PMC5035525

[13] Wen PY , Macdonald DR, Reardon DA, et al. Updated response assessment criteria for high-grade gliomas: response assessment in neuro-oncology working group. J Clin Oncol. 2010;28(11):1963–1972. 20231676 10.1200/JCO.2009.26.3541

[14] Ellingson BM , Wen PY, Cloughesy TF. Modified criteria for radiographic response assessment in glioblastoma clinical trials. Neurotherapeutics. 2017;14(2):307–320. 28108885 10.1007/s13311-016-0507-6 PMC5398984

[15] Brandes AA , Franceschi E, Tosoni A, et al. MGMT promoter methylation status can predict the incidence and outcome of pseudoprogression after concomitant radiochemotherapy in newly diagnosed glioblastoma patients. J Clin Oncol. 2008;26(13):2192–2197. 18445844 10.1200/JCO.2007.14.8163

[16] Li H , Li J, Cheng G, Zhang J, Li X. IDH mutation and MGMT promoter methylation are associated with the pseudoprogression and improved prognosis of glioblastoma multiforme patients who have undergone concurrent and adjuvant temozolomide-based chemoradiotherapy. Clin Neurol Neurosurg. 2016;151:31–36. 27764705 10.1016/j.clineuro.2016.10.004

[17] Lim YJ , Kim IH, Han TJ, et al. Hypofractionated chemoradiotherapy with temozolomide as a treatment option for glioblastoma patients with poor prognostic features. Int J Clin Oncol. 2015;20(1):21–28. 24705988 10.1007/s10147-014-0690-6

[18] Balana C , Capellades J, Pineda E, et al.; GLIOCAT Group. Pseudoprogression as an adverse event of glioblastoma therapy. Cancer Med. 2017;6(12):2858–2866. 29105360 10.1002/cam4.1242 PMC5727237

[19] Melguizo-Gavilanes I , Bruner JM, Guha-Thakurta N, Hess KR, Puduvalli VK. Characterization of pseudoprogression in patients with glioblastoma: is histology the gold standard? J Neurooncol. 2015;123(1):141–150. 25894594 10.1007/s11060-015-1774-5 PMC4780341

[20] Van Mieghem E , Wozniak A, Geussens Y, et al. Defining pseudoprogression in glioblastoma multiforme. Eur J Neurol. 2013;20(10):1335–1341. 23679051 10.1111/ene.12192

[21] Choi YJ , Kim HS, Jahng GH, Kim SJ, Suh DC. Pseudoprogression in patients with glioblastoma: added value of arterial spin labeling to dynamic susceptibility contrast perfusion MR imaging. Acta Radiol. 2013;54(4):448–454. 23592805 10.1177/0284185112474916

[22] Macdonald L , Cascino S, Clifford S, Cairncross JG. Response criteria for phase II studies of supratentorial malignant glioma. J Clin Oncol. 1990;8(7):1277–1280. 2358840 10.1200/JCO.1990.8.7.1277

[23] Mas-Moruno C , Rechenmacher F, Horst Kessler HC. The first anti-angiogenic small molecule drug candidate design, synthesis and clinical evaluation. Anticancer Agents Med Chem. 2010;10(10):753–768. 21269250 10.2174/187152010794728639 PMC3267166

[24] Kazazi-Hyseni F , Beijnen JH, Schellens JH. Bevacizumab. Oncologist. 2010;15(8):819–825. 20688807 10.1634/theoncologist.2009-0317 PMC3228024

[25] Rahmathulla G , Marko NF, Weil RJ. Cerebral radiation necrosis: a review of the pathobiology, diagnosis and management considerations. J Clin Neurosci. 2013;20(4):485–502. 23416129 10.1016/j.jocn.2012.09.011

[26] Furuse M , Nonoguchi N, Kawabata S, Miyatake S, Kuroiwa T. Delayed brain radiation necrosis: pathological review and new molecular targets for treatment. Med Mol Morphol. 2015;48(4):183–190. 26462915 10.1007/s00795-015-0123-2

[27] Dietrich J , Rao K, Pastorino S, Kesari S. Corticosteroids in brain cancer patients: benefits and pitfalls. Expert Rev Clin Pharmacol. 2011;4(2):233–242. 21666852 10.1586/ecp.11.1 PMC3109638

[28] Batchelor TT , Sorensen AG, di Tomaso E, et al. AZD2171, a pan-VEGF receptor tyrosine kinase inhibitor, normalizes tumor vasculature and alleviates edema in glioblastoma patients. Cancer Cell. 2007;11(1):83–95. 17222792 10.1016/j.ccr.2006.11.021 PMC2748664

[29] Brandsma D , van den Bent MJ. Pseudoprogression and pseudoresponse in the treatment of gliomas. Curr Opin Neurol. 2009;22(6):633–638. 19770760 10.1097/WCO.0b013e328332363e

[30] Wick W , Platten M, Wick A, et al. Current status and future directions of anti-angiogenic therapy for gliomas. Neuro Oncol. 2016;18(3):315–328. 26459812 10.1093/neuonc/nov180 PMC4767238

[31] Mezu-Ndubuisi OJ , Maheshwari A. The role of integrins in inflammation and angiogenesis. Pediatr Res. 2021;89(7):1619–1626. 33027803 10.1038/s41390-020-01177-9 PMC8249239

[32] Byzova T , Goldman C, Pampori N, et al. A mechanism for modulation of cellular responses to VEGF: activation of the integrins. Mol Cell. 2000;6(4):851–860. 11090623

[33] Somanath PR , Malinin NL, Byzova TV. Cooperation between integrin alphavbeta3 and VEGFR2 in angiogenesis. Angiogenesis. 2009;12(2):177–185. 19267251 10.1007/s10456-009-9141-9 PMC2863048

[34] Reynolds AR , Hart IR, Watson AR, et al. Stimulation of tumor growth and angiogenesis by low concentrations of RGD-mimetic integrin inhibitors. Nat Med. 2009;15(4):392–400. 19305413 10.1038/nm.1941

[35] Orecchia A , Lacal PM, Schietroma C, et al. Vascular endothelial growth factor receptor-1 is deposited in the extracellular matrix by endothelial cells and is a ligand for the alpha 5 beta 1 integrin. J Cell Sci. 2003;116(Pt 17):3479–3489. 12865438 10.1242/jcs.00673

[36] Bianconi D , Unseld M, Prager GW. Integrins in the spotlight of cancer. Int J Mol Sci . 2016;17(12):2037. 27929432 10.3390/ijms17122037 PMC5187837

[37] Wen P , van den Bent M, Youssef G, et al. RANO 2.0: update to the response assessment in neuro-oncology criteria for high- and low-grade gliomas in adults. J Clin Oncol. 2023;41(33):5187–5199. 37774317 10.1200/JCO.23.01059 PMC10860967

